# Comparative associations between anthropometric and bioelectric impedance analysis derived adiposity measures with blood pressure and hypertension in India: a cross-sectional analysis

**DOI:** 10.1186/s40608-017-0173-1

**Published:** 2017-12-01

**Authors:** Kevin Y. Taing, Michael E. Farkouh, Rahim Moineddin, Jack V. Tu, Prabhat Jha

**Affiliations:** 1 Centre for Global Health Research, St. Michael’s Hospital, Toronto, ON Canada; 2 Dalla Lana School of Public Health, University of Toronto, Toronto, ON Canada; 3 Peter Munk Cardiac Centre, University Health Network, Toronto, ON Canada; 4 The Heart and Stroke Richard Lewar Centre of Excellence in Cardiovascular Research, University of Toronto, Toronto, ON Canada; 5 Department of Family and Community Medicine, University of Toronto, Toronto, ON Canada; 6 Institute for Clinical Evaluative Sciences, Toronto, ON Canada; 7 Schulich Heart Centre, Sunnybrook Health Sciences Centre, Toronto, ON Canada; 8 Institute of Health Policy, Management and Evaluation, University of Toronto, Toronto, ON Canada

**Keywords:** Anthropometry, Bioelectrical impedance analysis, Adiposity, Body mass index, Waist circumference, Blood pressure, Hypertension, India

## Abstract

**Background:**

The utility of bioelectrical impedance analysis (BIA) derived adiposity measures as compared to anthropometric measures for the assessment of adiposity-related health risk is not clear. We aimed to clarify the relationships of BIA and anthropometric derived adipose measures with blood pressure and hypertension, and to compare the discriminative ability of the respective measures for hypertension.

**Methods:**

We used baseline data collected between 2015 and 2016 from the Indian Study on Health of Adults (ISHA), an ongoing population based cohort study in India (*N* = 5990; age 30–69 years). We examined and compared the associations and discriminative ability between anthropometric (body mass index, waist circumference, hip circumference, waist-hip ratio, waist-height ratio) and BIA (whole body and trunk fat percentage) derived adiposity measures with blood pressure components (systolic pressure, diastolic pressure, pulse pressure, mean arterial pressure, mid-blood pressure) and hypertension.

**Results:**

Regardless of whether the adiposity measure was derived from BIA or anthropometry, all were strongly and positively associated with blood pressure and hypertension. For both men and women, the magnitude of association of BIA measures with blood pressure and hypertension were comparable to those of anthropometric measures. Further, the ability of BIA derived adiposity measures to distinguish between those with and without hypertension was similar to the discriminative ability of anthropometric measures.

**Conclusions:**

As compared to simple anthropometric measures, BIA derived estimates of adiposity provide no apparent advantage in the assessment of blood pressure and hypertension. The observed similarities between adiposity measures suggest that simple anthropometrics may be sufficient to assess adiposity and adiposity-related risks.

## Background

In India, demographic, socioeconomic and health transitions have contributed to an increasing burden of adiposity-related diseases, such as cardiovascular disease (CVD) [1, 2]. A well-established consequence of excess adiposity is high blood pressure [3–5], which is a major determinant of CVD related morbidity and mortality [6–9]. Indeed, amongst CVD risk factors high blood pressure accounts for the greatest proportion of deaths in India [1]. Epidemiologic studies of the relationship between adiposity and blood pressure commonly use anthropometric indices [body mass index (BMI), waist circumference (WC), hip circumference (HC), waist-hip ratio (WHR), and waist-height ratio (WHtR)] as surrogate measures of adiposity. However, anthropometric measures are limited in their ability to distinguish differences in body composition, and do not provide estimates of body fat [10, 11]. As such, their adequacy to assess adiposity and adiposity-related health risks has been challenged, with inconsistent findings between studies [11–15].

Criterion methods such as magnetic resonance imaging (MRI) and dual-energy X-ray absorptiometry (DXA) are available to assess body composition and determine relative amounts of adipose tissue, but these methods are costly, invasive and time consuming. Moreover, they are not readily available for large scale use in low and middle-income countries (LMIC), such as India. In comparison, bioelectrical impedance analysis (BIA) represents a relatively low-cost and simple method to obtain estimates of body fat and identify those at increased health risk [16]. Further, with the advent of new BIA models capable of segmental analysis, assessment of whole body and regional (i.e. trunk) distribution of adipose tissue is feasible [17, 18]. The ability of BIA to distinguish differences in body composition and provide estimates of body fat may be of particular importance for a South Asian population. Compared to other ethnic groups, it has been shown that South Asians have higher amounts of body fat despite having similar or lower anthropometric values [19, 20]. As BIA provides estimates of body fat, it may prove to be more advantageous for the early detection and risk stratification of populations with high adiposity-related health risk than simple anthropometric measures in India. However, despite the potential benefits of BIA, there is a paucity of studies from India and other LMIC investigating its utility in assessing adiposity-related risks such as hypertension [21, 22].

As more sophisticated measures of body fat (i.e. those derived from BIA) become readily available, reliable studies evaluating whether they provide any value over simple anthropometric measures are necessary. We therefore examine and compare the associations of anthropometric and BIA derived adiposity measures with blood pressure and hypertension in a large population-based cohort of men and women from India.

## Methods

### Study design, setting and participants

We conducted a cross-sectional analysis comparing the associations between anthropometric and BIA derived adiposity measures with blood pressure and hypertension in India. The Indian Study on Health of Adults (ISHA) is an ongoing population-based cohort study of men and women age 30–69 years from the general population of the town of Barshi (Maharashtra, India). We used baseline data collected between 2015 and 2016 for our analyses (*N* = 5996). Of these participants, 6 were excluded because of pregnancy.

### Study data sources

Upon arrival to each village, and prior to the recruitment of participants, survey teams met with the local Sarpanch or authorized personal of the village to seek permission for the study. In addition, group meetings were also conducted before the start of the survey to create awareness of the study for those residing in the village community.

Trained surveyors collected data for the baseline survey in three stages over a 7-day activity cycle: enumeration (days 1–2), household health survey (days 3–4) and a health checkup camp (days 5–7). First, field teams enumerated the households in the village, collecting information on household size and the usual residing members. Second, trained surveyors (a male and a female) visited the households and interviewed eligible members (30–69 years of age) to obtain detailed information on demographic, socioeconomic, and lifestyle characteristics, along with blood pressure measurements and antihypertensive medication use. After completion of the interview participants were invited to attend a health checkup camp, and were given an invitation card detailing the time and place of their health checkup. Each health checkup camp was set up in the village with direct input from the Sarpanch or any other administrative head to ensure ease of access for participants. Lastly, participants attended the health checkup camp where their blood pressure was measured, along with physical and body composition measurements.

### Blood pressure measurement

After 5 min of rest, three blood pressure measurements were taken at heart level in a seated position using the Omron BP-742CAN (Kyoto, Japan) digital automatic monitor. Systolic blood pressure (SBP) and diastolic blood pressure (DBP) were calculated as the average of the three readings. We calculated pulse pressure, mean arterial pressure, and mid-blood pressure using previously reported formulas [23]. Hypertension was defined as SBP ≥140 or DBP ≥ 90, or reported use of antihypertensive medication.

### Physical measurements

A measuring tape was used to measure height to the nearest 0.1 cm [23]. Weight was measured to the nearest 0.1 kg using the Tanita MC780MA body composition analyzer (Tokyo, Japan), without footwear and with subtraction of 0.5 kg for clothing weight. WC and HC were both measured to the nearest 0.1 cm using a measuring tape. For WC, measurements were taken at the level of the umbilicus with arms folded across the chest. For HC, measurements were taken at the point yielding the maximum circumference over the buttocks.

### Adiposity measures

The anthropometric measures of adiposity were BMI, WC, HC, WHR, and WHtR. BMI was calculated as weight in kilograms divided by the square of height in meters. WHR was calculated as WC in centimeters divided by HC in centimeters, and WHtR as WC in centimeters divided by height in centimeters. The BIA derived measures of adiposity were whole body fat percentage and trunk fat percentage, as estimated with proprietary algorithms by the Tanita body composition analyzer.

### Statistical analysis

We performed all analyses separately for men and women. We calculated Pearson’s partial correlation coefficients for the intercorrelations of the different adiposity measures (BMI, WC, HC, WHR, WHtR, whole body fat percentage, trunk fat percentage) and blood pressure components (SBP, DBP, pulse pressure, mean arterial pressure, mid-blood pressure), adjusted for age. In order to compare the associations between anthropometric and BIA derived adiposity measures with blood pressure and hypertension, we evaluated the relationships using several methods. First, we used multiple linear regression models to quantify the associations between adiposity measures with blood pressure components. Second, we used Poisson regression models to examine the relationships between adiposity measures and hypertension. We estimated the means of each blood pressure component, and calculated the prevalence ratios (PR) for hypertension per sex-specific standard deviation (SD) change in an adiposity measure. We used two models for these analyses. In the first model, adjustments were made for age and level of education (illiterate, primary school, middle school, secondary school, college). In the second model, further adjustments were made variously for either an anthropometric or BIA derived measure of general (BMI, whole body fat percentage) or central (WC, trunk fat percentage) adiposity. 198 individuals (3% of total) who reported current use of antihypertensive medication were excluded from the continuous blood pressure analyses.

Lastly, we used receiver operating characteristic (ROC) curves and the area under the curve (AUC), estimated by logistic regression models with adjustments for age and education, to compare the discriminative ability of each adiposity measure for hypertension. The AUC is a measure of the overall discriminative ability of each adiposity measure for hypertension, with values falling between 0.5 and 1.0, representing no discriminative and perfect discriminative ability, respectively. The AUC for all measures of adiposity were compared using a nonparametric approach for the comparison of multiple AUC from ROC curves [24].

In addition to age and education adjustments, further adjustment for tobacco use (non-user, less than daily user, daily user) and alcohol consumption (non-drinker, current drinker) did not substantially affect any of the estimates (results not presented). We performed all statistical analyses using SAS version 9.3 (SAS Institute, Cary, NC, USA), and provide estimates with their respective 95% confidence intervals (CI).

## Results

The means (SD) of anthropometric and BIA derived adiposity measures, blood pressure components, and hypertension prevalence are presented in Table 1. The average age for men and women were 47 (12) and 45 (11) years, respectively, with the overall mean being 46 (11) years in our study. On average, SBP and pulse pressure were higher among men than women, whereas women had slightly higher DBP than men. The average mean arterial pressure, mid-blood pressure, and hypertension prevalence were similar between both sexes. In general, mean BMI and HC were slightly lower for men than women. Likewise, mean fat percentages (whole body and trunk) were also lower for men than women. By contrast, average WC, WHR and WHtR were greater for men than women.

**Table 1:** Distribution of adiposity measures and blood pressure (*N* = 5990)

	Male	Female
	No. 2764	No. 3226
Age	47.1 (11.7)	45.2 (11.1)
Anthropometric Measures		
Height (cm)	163.4 (6.3)	150.0 (5.7)
Weight (kg)	60.0 (11.4)	51.0 (10.0)
Body Mass Index (kg/m^2^)	22.4 (3.7)	22.7 (4.1)
Waist Circumference (cm)	82.5 (11.1)	73.5 (10.1)
Hip Circumference (cm)	90.0 (7.2)	92.1 (8.5)
Waist-Hip Ratio	0.91 (0.075)	0.80 (0.064)
Waist-Height Ratio	0.50 (0.067)	0.49 (0.068)
Bioelectrical Impedance Measures		
Whole Body Fat (%)	20.0 (6.4)	32.0 (7.4)
Trunk Fat (%)	20.6 (7.8)	31.0 (9.2)
Blood Pressure Components (mmHg)		
Systolic Blood Pressure	129.0 (16.5)	127.8 (18.4)
Diastolic Blood Pressure	83.0 (9.6)	83.6 (9.6)
Pulse Pressure	46.0 (10.8)	44.2 (12.7)
Mean Arterial Pressure	98.3 (11.3)	98.3 (11.8)
Mid-Blood Pressure	106.0 (12.4)	105.7 (13.3)
Hypertension (%)^a^	803 (29.1)	984 (30.5)

Values presented as mean (SD), unless indicated. ^a^No. (%) hypertensive

Overall, the sex-specific correlation coefficients of adiposity measures with blood pressure components were comparable, except for pulse pressure, whereby the correlations were relatively weaker (Table 2). The intercorrelations between anthropometric measures were high, apart from those with WHR, which were relatively lower. Similarly, BIA derived measures of adiposity were also strongly correlated with each other. When considering the pairwise correlation coefficients between anthropometric and BIA derived adiposity measures, BMI had the strongest correlation with body fat and trunk fat percentage for both men and women, whereas WHR had the weakest.

**Table 2:** Pearson partial correlation coefficients adjusted for age (*N* = 5792)

	BMI	WC	HC	WHR	WHtR	Whole Body Fat	Trunk Fat	SBP	DBP	PP	MAP	MBP
BMI	Male:	0.90	0.85	0.64	0.90	0.82	0.82	0.29	0.28	0.18	0.30	0.30
	Female:	0.88	0.90	0.47	0.88	0.90	0.93	0.24	0.32	0.09	0.30	0.29
WC		Male:	0.83	0.83	0.96	0.80	0.80	0.27	0.29	0.15	0.29	0.29
		Female:	0.84	0.76	0.96	0.82	0.85	0.24	0.32	0.09	0.30	0.28
HC			Male:	0.39	0.76	0.73	0.74	0.24	0.24	0.15	0.25	0.25
			Female:	0.28	0.77	0.83	0.86	0.21	0.29	0.07	0.27	0.25
WHR				Male:	0.84	0.60	0.60	0.21	0.24	0.10	0.24	0.23
				Female:	0.76	0.46	0.48	0.17	0.21	0.07	0.20	0.19
WHtR					Male:	0.79	0.80	0.27	0.28	0.15	0.29	0.29
					Female:	0.83	0.85	0.25	0.32	0.10	0.31	0.29
Whole Body Fat						Male:	0.97	0.30	0.32	0.17	0.33	0.32
						Female:	0.97	0.23	0.31	0.07	0.29	0.27
Trunk Fat							Male:	0.30	0.32	0.15	0.33	0.32
							Female:	0.23	0.32	0.08	0.30	0.28
SBP								Male:	0.79	0.81	0.93	0.97
								Female:	0.77	0.84	0.93	0.97
DBP									Male:	0.28	0.96	0.91
									Female:	0.30	0.95	0.90
PP										Male:	0.55	0.64
										Female:	0.59	0.68
MAP											Male:	0.99
											Female:	0.99

Correlations exclude those on antihypertensive medication. All correlations significant at *p* < 0.0001. *BMI* body mass index, *WC* waist circumference, *HC* hip circumference, *WHR* waist-hip ratio, *WHtR* waist-height ratio, *SBP* systolic blood pressure, *DBP* diastolic blood pressure, *PP* pulse pressure, *MAP* mean arterial pressure, *MBP* mid-blood pressure

### Adiposity measures and blood pressure

The differences in mean blood pressure components [mmHg per SD (95% CI)] for each anthropometric and BIA derived adiposity measure are presented in Table 3. Irrespective of whether the measures of adiposity were calculated from anthropometry or BIA, all measures were strongly and positively associated with most blood pressure components for both men and women. The strongest associations were between adiposity measures and SBP, whereas the weakest relationships were observed with pulse pressure.

**Table 3:** Mean differences in blood pressure for adiposity measures (N = 5792)

	mmHg per SD (95% CI)									
Measurement (SD)	SBP		DBP		PP		MAP		MBP	
Male										
Model 1										
Body Mass Index (3.73 kg/m^2^)	4.56	(3.98, 5.14)	2.71	(2.36, 3.07)	1.85	(1.46, 2.23)	3.33	(2.93, 3.74)	3.64	(3.20, 4.08)
Waist Circumference (11.13 cm)	4.17	(3.59, 4.76)	2.71	(2.36, 3.06)	1.46	(1.08, 1.85)	3.20	(2.79, 3.60)	3.44	(3.00, 3.88)
Hip Circumference (7.20 cm)	3.76	(3.18, 4.35)	2.27	(1.91, 2.62)	1.50	(1.11, 1.88)	2.77	(2.36, 3.17)	3.02	(2.57, 3.46)
Waist-Hip Ratio (0.075)	3.23	(2.64, 3.82)	2.25	(1.89, 2.61)	0.98	(0.59, 1.37)	2.58	(2.16, 2.99)	2.74	(2.29, 3.19)
Waist-Height Ratio (0.067)	4.21	(3.63, 4.80)	2.69	(2.34, 3.05)	1.52	(1.14, 1.91)	3.20	(2.79, 3.60)	3.45	(3.01, 3.89)
Whole Body Fat (6.44%)	4.69	(4.11, 5.27)	3.02	(2.67, 3.36)	1.68	(1.29, 2.06)	3.57	(3.18, 3.97)	3.85	(3.42, 4.29)
Trunk Fat (7.82%)	4.62	(4.04, 5.20)	3.07	(2.72, 3.42)	1.55	(1.17, 1.94)	3.59	(3.19, 3.99)	3.85	(3.41, 4.28)
Model 2										
Body Mass Index (3.73 kg/m^2^)^a^	2.13	(1.12, 3.13)	0.71	(0.10, 1.32)	1.42	(0.75, 2.08)	1.18	(0.49, 1.88)	1.42	(0.66, 2.18)
Whole Body Fat (6.44%)^b^	2.96	(1.96, 3.96)	2.44	(1.83, 3.04)	0.52	(−0.14, 1.19)	2.61	(1.92, 3.30)	2.70	(1.95, 3.46)
Waist Circumference (11.13 cm)^c^	1.33	(0.37, 2.30)	0.71	(0.13, 1.29)	0.62	(−0.02, 1.26)	0.92	(0.25, 1.59)	1.02	(0.29, 1.75)
Trunk Fat (7.82%)^d^	3.55	(2.59, 4.52)	2.50	(1.92, 3.08)	1.05	(0.41, 1.70)	2.85	(2.18, 3.52)	3.03	(2.29, 3.76)
Female										
Model 1										
Body Mass Index (4.06 kg/m^2^)	4.00	(3.43, 4.57)	3.02	(2.70, 3.33)	0.98	(0.59, 1.38)	3.35	(2.97, 3.72)	3.51	(3.09, 3.93)
Waist Circumference (10.09 cm)	4.00	(3.43, 4.58)	3.07	(2.75, 3.39)	0.94	(0.54, 1.33)	3.38	(3.00, 3.76)	3.53	(3.11, 3.96)
Hip Circumference (8.51 cm)	3.47	(2.90, 4.04)	2.76	(2.44, 3.08)	0.71	(0.32, 1.10)	3.00	(2.62, 3.37)	3.11	(2.69, 3.54)
Waist-Hip Ratio (0.064)	2.86	(2.27, 3.46)	2.06	(1.72, 2.39)	0.81	(0.40, 1.21)	2.33	(1.93, 2.72)	2.46	(2.02, 2.90)
Waist-Height Ratio (0.068)	4.26	(3.68, 4.84)	3.12	(2.80, 3.44)	1.14	(0.74, 1.53)	3.50	(3.12, 3.88)	3.69	(3.27, 4.11)
Whole Body Fat (7.38%)	3.73	(3.17, 4.30)	2.94	(2.63, 3.26)	0.79	(0.40, 1.18)	3.21	(2.83, 3.58)	3.34	(2.92, 3.76)
Trunk Fat (9.23%)	3.86	(3.29, 4.42)	3.04	(2.73, 3.35)	0.82	(0.43, 1.21)	3.31	(2.94, 3.69)	3.45	(3.03, 3.86)
Model 2										
Body Mass Index (4.06 kg/m^2^)^a^	3.31	(2.00, 4.61)	1.89	(1.16, 2.61)	1.42	(0.52, 2.32)	2.36	(1.50, 3.22)	2.60	(1.64, 3.55)
Whole Body Fat (7.38%)^b^	0.77	(−0.53, 2.07)	1.25	(0.53, 1.98)	−0.48	(−1.38, 0.41)	1.09	(0.23, 1.95)	1.01	(0.06, 1.96)
Waist Circumference (10.09 cm)^c^	2.41	(1.32, 3.50)	1.58	(0.98, 2.18)	0.83	(0.09, 1.58)	1.85	(1.14, 2.57)	1.99	(1.20, 2.79)
Trunk Fat (9.23%)^d^	1.85	(0.78, 2.91)	1.73	(1.14, 2.32)	0.12	(−0.61, 0.86)	1.77	(1.06, 2.47)	1.79	(1.01, 2.57)

Estimates exclude those on antihypertensive medication. Model 1, estimates from multiple linear regression, adjusted for age and education. Model 2, model 1 plus additional adjustments for ^a^whole body fat percentage, ^b^body mass index, ^c^trunk fat percentage, ^d^waist circumference. *SBP* systolic blood pressure, *DBP* diastolic blood pressure, *PP* pulse pressure, *MAP* mean arterial pressure, *MBP* mid-blood pressure, *SD* standard deviation, *CI* confidence interval

On average, the associations between anthropometric derived measures and blood pressure were slightly weaker than BIA derived measures among men. For instance, each SD difference in an anthropometric measure was associated with a 3.23 mmHg to 4.56 mmHg change in SBP, whereas changes in SBP for each SD difference in a BIA measure ranged from 4.62 to 4.69 mmHg. In comparison, the relationship of anthropometric measures with SBP were moderately stronger than BIA measures among women, and ranged from 2.86 to 4.26 mmHg, and 3.73 mmHg to 3.86 mmHg for anthropometric and BIA derived measures, respectively. Apart from pulse pressure, which had much weaker associations with all measures of adiposity, similar (albeit somewhat weaker) relationships were observed between each measure and the remaining blood pressure components.

The further adjustment of an anthropometric measure for a BIA measure, and vice versa, diminished the relation between each (general or central) adiposity measure and all blood pressure components regardless of measurement method. Although attenuated, the association between both an anthropometric and BIA derived measure with blood pressure were, for the most part, independent of each other and remained significant (*p* < 0.05).

### Adiposity measures and hypertension

**Fig. 1:**
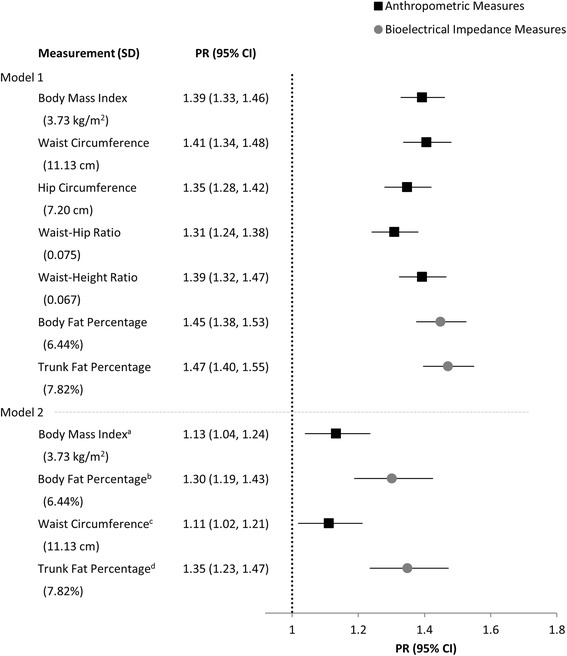
Prevalence ratios for hypertension per SD of each adiposity measure among men (*N* = 2764). Model 1, estimates from Poisson regression, adjusted for age and education. Model 2, model 1 plus additional adjustments for ^a^whole body fat percentage, ^b^body mass index, ^c^trunk fat percentage, ^d^waist circumference. PR, prevalence ratio; SD, standard deviation; CI, confidence interval

**Fig. 2:**
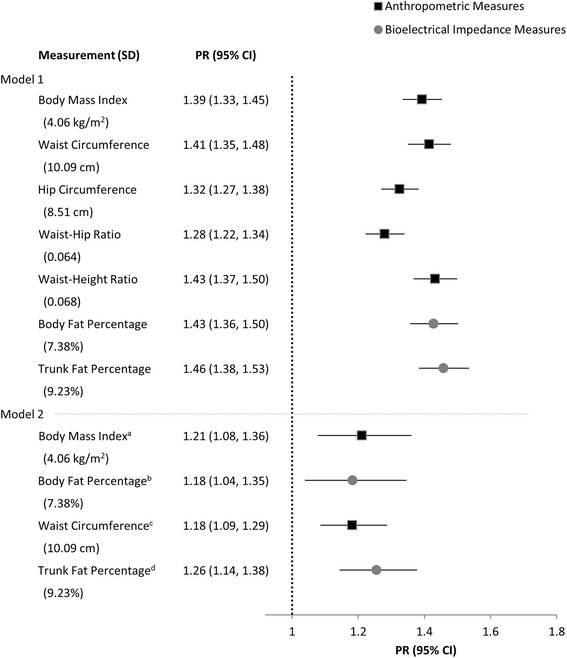
Prevalence ratios for hypertension per SD of each adiposity measure among women (*N* = 3226). Model 1, estimates from Poisson regression, adjusted for age and education. Model 2, model 1 plus additional adjustments for ^a^whole body fat percentage, ^b^body mass index, ^c^trunk fat percentage, ^d^waist circumference. PR, prevalence ratio; SD, standard deviation; CI, confidence interval

The PRs (95% CI) for hypertension per SD difference in each measure of adiposity are shown for men and women in Figs. 1 and 2, respectively. Overall, the relationships between either an anthropometric or BIA derived adiposity measure and hypertension were similar. The weakest associations were observed between WHR and hypertension for both men and women [PR: 1.31 (1.24, 1.38) and 1.28 (1.22, 1.34), respectively], whereas the strongest associations were for trunk fat percentage [PR: 1.47 (1.40, 1.55) and 1.46 (1.38, 1.53), respectively]. Nevertheless, regardless of whether the measure of adiposity was calculated from anthropometric or BIA derived measures, the relative difference between them for their relationship with hypertension were slight. For instance, the difference between the anthropometric and BIA measure that had the strongest relationship with hypertension was less than 5% for both sexes.

The further adjustment of an anthropometric measure for a BIA measure, and vice versa, resulted in an attenuation of their relationships with hypertension. Among men, there appears to be a more marked attenuation of the association of BMI and WC with hypertension after adjusting for body and trunk fat percentage, respectively (Fig. 1, Model 2). Nonetheless, despite being diminished, all adiposity measures remained significantly (*p* < 0.05) associated with hypertension regardless of whether or not additional adjustments were made for the other measurement method.

### Adiposity measures and ROC curves, AUC for hypertension

Summarized in Table 4 are the ROC curves AUC (95% CI) of each adiposity measure for the discrimination of hypertension. For men and women, all adiposity measures regardless of whether an anthropometric or BIA derived measure demonstrated significant discriminative ability for hypertension, with AUCs ranging from 0.68 to 0.76. Except for HC and WHR, the AUC of each anthropometric measure was similar to the AUC of BIA derived measures for both sexes. However, in comparison to whole body and trunk fat percentage, the AUC for HC and WHR were significantly (*p* < 0.05) less for both sexes. Nevertheless, the relative sex-specific difference for the AUC between either an anthropometric or BIA derived measure of adiposity was negligible, and not statistically significant between the majority of measures irrespective of their derivation method.

**Table 4:** Area under the receiver operating characteristic curves (N = 5990)

	Male	Female
	Area Under Curve (95% CI)	
Anthropometric Measures		
Body Mass Index	0.70 (0.68, 0.72)	0.76 (0.74, 0.77)
Waist Circumference	0.70 (0.68, 0.72)	0.76 (0.74, 0.77)
Hip Circumference	0.69 (0.66, 0.71)	0.75 (0.73, 0.76)
Waist-Hip Ratio	0.68 (0.66, 0.70)	0.73 (0.72, 0.75)
Waist-Height Ratio	0.70 (0.68, 0.72)	0.76 (0.74, 0.78)
Bioelectrical Impedance Measures		
Body Fat Percentage	0.71 (0.68, 0.73)	0.75 (0.74, 0.77)
Trunk Fat Percentage	0.71 (0.69, 0.73)	0.76 (0.74, 0.77)
	Difference Between Area Under Curve (95% CI)	
Body Fat Percentage^a^		
Body Mass Index	−0.0046 (−0.0143, 0.0051)	0.0021 (−0.0035, 0.0077)
Waist Circumference	−0.0067 (−0.0171, 0.0037)	0.0010 (−0.0062, 0.0082)
Hip Circumference	−0.0194 (−0.0312, −0.0076)	−0.0083 (−0.0150, −0.0016)
Waist-Hip Ratio	−0.0242 (−0.0383, −0.0101)	−0.0193 (−0.0305, −0.0082)
Waist-Height Ratio	−0.0088 (−0.0192, 0.0015)	0.0048 (−0.0025, 0.0121)
Trunk Fat Percentage	0.0026 (−0.0018, 0.0069)	0.0019 (−0.0020, 0.0058)
Trunk Fat Percentage^b^		
Body Mass Index	−0.0072 (−0.0168, 0.0024)	0.0002 (−0.0039, 0.0043)
Waist Circumference	−0.0093 (−0.0193, 0.0008)	−0.0009 (−0.0071, 0.0054)
Hip Circumference	−0.0219 (−0.0337, −0.0102)	−0.0102 (−0.0160, −0.0043)
Waist-Hip Ratio	−0.0267 (−0.0408, −0.0127)	−0.0212 (−0.0323, −0.0101)
Waist-Height Ratio	−0.0114 (−0.0215, −0.0013)	0.0029 (−0.0035, 0.0093)
Body Fat Percentage	−0.0026 (−0.0069, 0.0018)	−0.0019 (−0.0058, 0.0020)

Area under the receiver operating characteristic curves for the discriminative ability of adiposity measures for hypertension, adjusted for age and education. ^a^Differences between anthropometric measures and whole body fat percentage area under curve, ^b^differences between anthropometric measures and trunk fat percentage area under curve

## Discussion

In our study of adults from India, the strength of association of anthropometric (BMI, WC) and BIA (whole body and trunk fat percentage) derived adiposity measures with blood pressure and hypertension were similar. Moreover, the discriminative ability of BIA measures for hypertension was comparable to anthropometric measures. Our findings suggest that BIA derived measures of adiposity have no apparent benefit over simple anthropometrics in the assessment of blood pressure and hypertension.

Although there is potential for the utilization of BIA to assess body composition and health risks, there is a paucity of studies from India reporting its use. Further, very limited information is available on the association between BIA derived body fat and health risks related to CVD. One study among 850 men in India indicates that high body fat percentage, as determined by BIA, is associated with hypertension and high coronary artery disease prevalence [22]. Similarly, we found that body fat percentage was positively associated with blood pressure and hypertension. However, to the best of our knowledge, no study has investigated the relative associations of both anthropometric and BIA derived measures of adiposity with blood pressure and hypertension in India. As such, our study is the first to describe these relationships and further studies in India are necessary for comparison purposes. Nevertheless, considered together with studies conducted elsewhere [14, 15], our findings provide additional support for the adequacy of anthropometric measures to assess adiposity-related health risks.

An often cited limitation of BMI and anthropometric measures in general, is their inability to discern differences in body composition [10]. Hence, the use of BIA is an appealing alternative as it is able to provide estimates of separate body tissues. In spite of this advantage, we only observed modest differences between the associations of anthropometric and BIA derived adiposity measures with blood pressure and hypertension. In particular, the relations of BIA measures were slightly stronger than anthropometric measures among men. By contrast, the associations of BIA measures were slightly weaker for women. The weaker relationships for women may be due, in part, to some imprecision in the estimation of adiposity by BIA among women. For instance, it has been suggested that the relative volume of adipose to muscle tissue may affect BIA. Specifically, greater adipose than muscle tissue among women may affect the calculated resistance by BIA, thus having an effect on the estimated body composition [25]. Future studies considering the use of BIA or anthropometric measures to investigate the association between adiposity and CVD should take into consideration these potential sex-related differences in their associations with blood pressure.

The capability of segmental analysis by BIA may be a useful addition to whole body analysis as it is able to provide information on the distribution of body tissues. However, given our findings, no further value of BIA was obtained from the use of segmental analysis for investigating the association of adiposity with blood pressure and hypertension. Both whole body and trunk fat estimates were highly correlated with each other (i.e. correlation coefficient of 0.97), which may explain, to some extent, the similarities in their observed relationships. Indeed, the use of segmental analysis may be limited by its ability to accurately assess different body components as compared to the body as a whole. For example, using DXA as the reference, it has been shown that estimates of segments (arms, legs, and trunk) may be more prone to error than estimation of whole body composition by BIA [17]. Taking this into consideration, and given the comparability between estimated whole body and trunk fat in our study, the use of a whole body BIA device may be sufficient. Further studies in India are required to investigate whether low-cost whole body BIA devices perform equally well as the more expensive segmental devices.

Apart from HC and WHR, we found no significant differences for the discriminative ability between anthropometric and BIA derived measures of adiposity for hypertension. In comparison to BIA estimated whole body and trunk fat, HC and WHR had significantly lower discriminative power for hypertension. This may be due, in part, to the limited ability of HC alone to distinguish those with greater adiposity, and the potential for decreased precision of WHR as a measure of adiposity. For instance, WHR does not differentiate those with either a small WC and HC or a large WC and HC, both would have a similar WHR. The inability to distinguish these differences may diminish the relationship that WHR has with blood pressure and hypertension, and indeed, among all the adiposity measures, the weakest associations were most consistently observed for WHR. Hence, our results suggest that HC and WHR may have lesser value than the other anthropometric measures considered in our study for assessing hypertension risk. Nonetheless, comparable to the similarities found for the strength of association between anthropometric and BIA measures with blood pressure and hypertension, the overall differences in discriminative ability between all measures were slight.

The limitations of our study warrant mention. First, the generalizability of our findings may be limited by the population-based study. Second, due to the cross-sectional analysis, causal inferences cannot be made for the association of adiposity with blood pressure and hypertension. However, the largely causal relationship between adiposity and blood pressure is well-established [3], and the reverse is implausible. Third, although the use of BIA is a more feasible method than DXA or MRI to derive estimates of body composition, the accuracy of estimated body fat may depend partly on the specific algorithm and equation used by a BIA device. Nevertheless, there is evidence from India demonstrating the likely validity of BIA derived measures as compared to DXA [21]. Further, BIA is the most practical and feasible method to obtain quick estimates of body composition in resource-limited settings. Notwithstanding these limitations, to our knowledge, our study is the first to systematically investigate the utility of BIA estimates of adiposity as compared to simple anthropometric measures in India. Additionally, our findings provide further insight and guidance for future studies considering the use of anthropometric or BIA derived measures to examine adiposity-related health risks in India.

## Conclusion

We provide additional evidence to support the adequacy of simple anthropometric measures in evaluating adiposity-related risk. Our findings suggest no material differences between BIA and anthropometric measures of adiposity in relation with blood pressure and hypertension. The observed similarities between adiposity measures may have important implications for facilitating research and risk stratification of populations in remote resource-limited settings where more intricate health measures are not feasible. Nevertheless, as more sophisticated body composition methods become readily available, further studies in India and other LMIC are required to identify the best and most suitable measure of adiposity and its associated risks.

## Abbreviations

AUC: Area under curve
BIA: Bioelectrical impedance analysis
BMI: Body mass index
CI: Confidence interval
CVD: Cardiovascular disease
DBP: Diastolic blood pressure
DXA: Dual-energy X-ray absorptiometry
HC: Hip circumference
ISHA: Indian study on health of adults
LMIC: Low and middle-income countries
MRI: Magnetic resonance imaging
PR: Prevalence ratio
ROC: Receiver operating characteristic
SBP: Systolic blood pressure
SD: Standard deviation
WC: Waist circumference
WHR: Waist-hip ratio
WHtR: Waist-height ratio
